# Influence of Precursor Composition on Microstructure Formation in Protein-Derived Porous Graphitic Aerogels

**DOI:** 10.1021/acsmaterialsau.5c00227

**Published:** 2026-01-27

**Authors:** M. Shaharyar Wani, Elizabeth G. Stump, Bridget R. Denzer, Craig B. Arnold

**Affiliations:** † Department of Mechanical & Aerospace Engineering, 6740 Princeton University, Princeton, New Jersey 08544, United States; ‡ Princeton Materials Institute, Princeton University, Princeton, New Jersey 08544, United States; § Department of Materials Science & Engineering, Massachusetts Institute of Technology, Cambridge, Massachusetts 02139, United States

**Keywords:** proteins, pyrolysis, carbon aerogel, hierarchically porous, graphitic

## Abstract

Hierarchically porous graphitic aerogels (HGAs) are promising carbon materials owing to their low density, multilevel porosity, and interconnected frameworks, making them suitable for applications in energy storage, water purification, and environmental remediation. In this work, HGAs were synthesized from a range of protein-based precursors, including pasteurized egg white (PEW), α-lactalbumin, β-lactoglobulin, bovine serum albumin (BSA), and yogurt, through pyrolysis. The study investigates how precursor composition influences thermal decomposition and microstructural development. All precursors except yogurt formed interconnected sheet- and fiber-like frameworks due to the self-foaming effect during pyrolysis. Despite its high protein content, the yogurt precursor produced a rough porous morphology, likely due to the presence of fats and inorganic species that inhibit foaming action. Elemental analysis confirmed the presence of significant amounts of inorganic species, such as calcium and phosphorus, in the yogurt-derived samples. To examine the influence of nonprotein constituents, a control experiment was conducted using a casein–whey mixture, the primary protein component of yogurt. Pyrolysis of this mixture produced aerogels with interconnected sheet- and fiber-like morphologies, confirming that the additional nonprotein components in yogurt affect structure formation. Further Raman analysis indicates that increasing the pyrolysis temperature enhanced carbonization and graphitic ordering across all the samples. Overall, this work provides insight into developing HGAs from diverse protein precursors and shows how precursor composition influences microstructural development during protein pyrolysis, offering valuable guidance for the design of HGA-based composites and functional materials.

## Graphical abstract

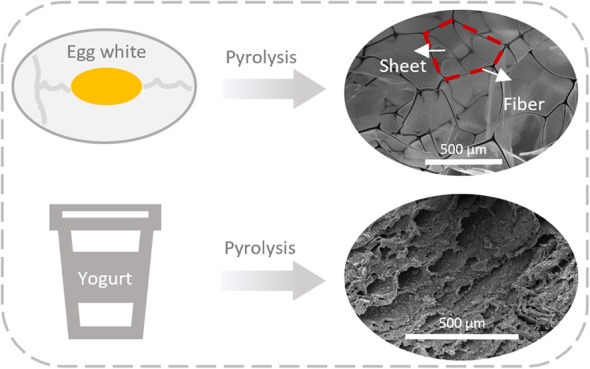

## Introduction

Carbon aerogels are solid, lightweight, and highly porous materials composed of more than 90% air by volume. Their unique three-dimensional interconnected network imparts a combination of desirable physicochemical properties, including ultralow density, high specific surface area, efficient mass transport, and low thermal conductivity.^1–3^ These characteristics position aerogels as attractive candidates for diverse applications in energy storage, water purification, and environmental remediation.^4–13^ However, the fabrication of these materials often relies on nonrenewable precursors and requires multistep synthesis, templating, and harsh chemical reagents, all of which pose significant challenges for their processing.

In recent studies from our group, we demonstrated that hierarchically porous graphitic aerogels (HGAs) with integrated sheet- and fiber-like morphologies can be synthesized through the pyrolysis of egg white protein.^14,15^ This process relies on an intrinsic self-foaming effect that emerges during controlled thermal decomposition, eliminating the need for any external foaming agents or templating additives. Although this approach does not involve a classical gel phase, self-foaming during pyrolysis yields an ultralight, three-dimensionally interconnected porous carbon network with bulk densities as low as ∼3.62 mg cm^–3^ and high porosity, consistent with established structural definitions of carbon-based aerogels.^15^ Owing to their distinctive hierarchical architecture, which combines ultralow density, high specific surface area, and multiscale porosity, these materials exhibit promising performance in a range of applications, including supercapacitors, electromagnetic interference shielding, photothermal depolymerization, water desalination, and microplastic removal.^14,16–18^ While our previous work established egg white as a model system for probing formation mechanisms, and protein composition has been widely studied in the context of wet foam formation under ambient conditions, the role of protein precursor composition in governing self-foaming behavior and solid-state microstructure formation during pyrolysis remains largely unexplored. In this study, we investigate the formation of HGAs from a range of protein-based precursors, including pasteurized egg white (PEW), α-lactalbumin, β-lactoglobulin, bovine serum albumin (BSA), and yogurt, through pyrolysis. The work focuses on understanding how differences in precursor composition influence thermal decomposition and microstructure development. Microscopy analyses of the pyrolyzed samples show that all protein precursors except yogurt form interconnected sheet- and fiber-like morphologies. Although yogurt contains a substantial protein fraction, its composition also includes fat and inorganic species, which appear to hinder the formation of a sheet- and fiber-like framework. Elemental analysis confirmed the presence of significant amounts of inorganic species such as calcium and phosphorus in the yogurt-derived samples. To examine the influence of nonprotein constituents, a control experiment was conducted using a casein–whey mixture, the primary protein component of yogurt. Pyrolysis of this mixture produced aerogels with interconnected sheet- and fiber-like morphologies, confirming that the additional nonprotein components in yogurt affect microstructure formation. Further, Raman analysis showed that increasing the pyrolysis temperature enhanced carbonization and graphitic ordering across all the samples. Together, these results provide insight into developing HGAs from diverse protein precursors and investigate how precursor composition influences microstructural evolution during protein pyrolysis, offering useful guidance for the design of HGA-based composites and functional materials.

## Experimental Section

### Materials

Lyophilized α-lactalbumin, β-lactoglobulin, and BSA were acquired from Millipore Sigma. These materials were used as received, without any chemical modification. PEW and yogurt were purchased from a local grocery store and were then lyophilized.

### Synthesis of HGA

Figure 1a presents a schematic of the synthesis process for HGAs from the various protein precursors. Commercially sourced PEW and yogurt were freeze-dried, while α-lactalbumin, β-lactoglobulin, and BSA were obtained in freeze-dried form. Freeze-drying was employed in this study to ensure consistent precursor processing (drying); however, it is not a prerequisite for material synthesis, as discussed in prior work.^15^ The precursors were placed in an alumina boat and pyrolyzed in a tube furnace under nitrogen, using a heating rate of 35 °C min^–1^ to peak temperatures of 250, 400, and 900 °C.^14^ The samples were held isothermally at 900 °C for 24 h to ensure complete carbonization and then cooled to room temperature under continuous nitrogen flow.

**Figure 1:**
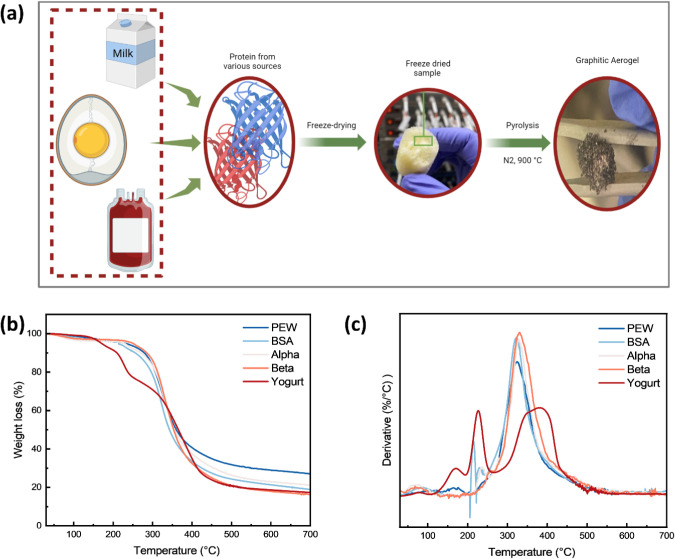
(a) Schematic diagram showing the synthesis of HGA from various protein precursors. Thermal analysis of different precursors (b) TGA profiles. (c) DTG curves.

### Material Characterizations

The surface morphologies of the synthesized samples were examined by environmental scanning electron microscopy (ESEM) using a Quanta 200 FEG instrument. Elemental analysis was performed using energy-dispersive X-ray spectroscopy (EDS) coupled to the ESEM. Sample density (ρ) was determined from the measured mass (*m*) and volume (*V*) using ρ *= m/V*. Raman spectroscopic analysis of the samples was conducted using a 532 nm laser excitation on a Horiba Raman spectrometer. TGA analysis was run using a PerkinElmer TGA-GC/MS system in thermogravimetric mode with heating from room temperature to 700 °C and 900 °C at 20 °C/min under a nitrogen atmosphere. Differential Scanning Calorimeter (DSC) analysis was carried out at a heating rate of 5 °C/min to 350 °C using PerkinElmer DSC-8500. Infrared thermal imaging and temperature measurements were conducted using a TC004 thermographic camera (Topdon, USA).

## Results and Discussion

The thermal decomposition behavior of protein precursors provides an essential insight into the transformations that occur during pyrolysis. To characterize these transitions, thermogravimetric analysis (TGA), derivative thermogravimetry (DTG) and differential scanning calorimetry (DSC) were performed on PEW, α-lactalbumin, β-lactoglobulin, BSA, and yogurt, as shown in Figure 1b,c and Supplementary Figure S1. Figure 1b presents the mass-loss behavior of the protein-based precursors, which exhibit predominantly three distinct decomposition regions. The first region, occurring between approximately 30 and 200 °C, corresponds to the loss of adsorbed and bound water.^19^ This is supported by the DSC results (Supplementary Figure S1), which show a broad endothermic peak in the same temperature range. The second region, occurring between approximately 200 and 450 °C, exhibits a large mass loss associated with the thermal decomposition of the protein precursors.^20^ This stage is also reflected in the DSC trace, which shows a broad endothermic peak at ∼250 °C. In this stage, polypeptide chains undergo cleavage through deamination, decarboxylation, and side-chain breakdown, generating volatile species such as CO_2_, CO, and small organic fragments. These reactions collapse the native protein structure and initiate the transition toward a carbon-rich char-like residue. The sharp mass loss observed around 320 °C, indicated by the steep slopes in the DTG curves (Figure 1c), corresponds to the peak rate of decomposition. In the third region, occurring beyond ∼450 °C, the char-like residue undergoes further reduction and carbonizes and develops some graphitic ordering. Thermogravimetric analysis performed up to 900 °C under nitrogen shows a char yield of ∼20.1%, providing a quantitative measure of carbon yield from the protein precursor (Supplementary Figure S3).

As described above, TGA revealed three distinct regions of mass loss during the thermal decomposition of the protein precursors, corresponding to dehydration, decomposition of organic constituents, and carbonization. In the 200 to 450 °C range, a large evolution of gaseous species, including CO_2_, CO and SO_2_ occurs. The generation and movement of these gases within the softened char-like residue create internal pressures that promote bubble formation and structural rearrangement. Previous studies have shown that PEW undergoes pronounced expansion during pyrolysis due to this gas-driven foaming process.^15^ To determine whether similar behavior occurs in other protein precursors and to assess the role of gas evolution in morphology development, scanning electron microscopy was performed on samples pyrolyzed at peak processing temperatures of 250 °C, 400 °C, and 900 °C. These temperatures correspond to stages before, during, and after the main decomposition event, allowing direct visualization of the morphological transition from the protein-based precursor to the carbonized graphitic material.

The morphological evolution of the protein precursors at different peak pyrolysis temperatures is presented in Figure 2. Figure 2a–e corresponds to samples pyrolyzed at 250 °C, Figure 2f–j at 400 °C, and Figure 2k–o at 900 °C, representing conditions before, during, and after the main decomposition events. At 250 °C, samples prepared from PEW, α-lactalbumin, β-lactoglobulin and BSA exhibit flaky surface morphologies (Figure 2a–d). In contrast, the sample prepared from yogurt precursor shows a particle-like structure composed of irregularly shaped aggregates (Figure 2e). In samples prepared at 400 °C, noticeable changes in morphology are observed for PEW, α-lactalbumin, β-lactoglobulin and BSA precursors, displaying the presence of interconnected sheet- and fiber-like structures (Figure 2f–i). In contrast, the yogurt-derived sample retains a particle-like morphology similar to that observed at 250 °C (Figure 2j). At 900 °C, PEW, α-lactalbumin, β-lactoglobulin and BSA samples retain the sheet- and fiber-like morphologies observed at 400 °C, with the sheets appearing more continuous and better defined (Figure 2k–n). In contrast, the yogurt-derived sample exhibits a distinctly different morphology, characterized by a porous, rough surface without any presence of sheet- or fiber-like formations (Figure 2o).

**Figure 2:**
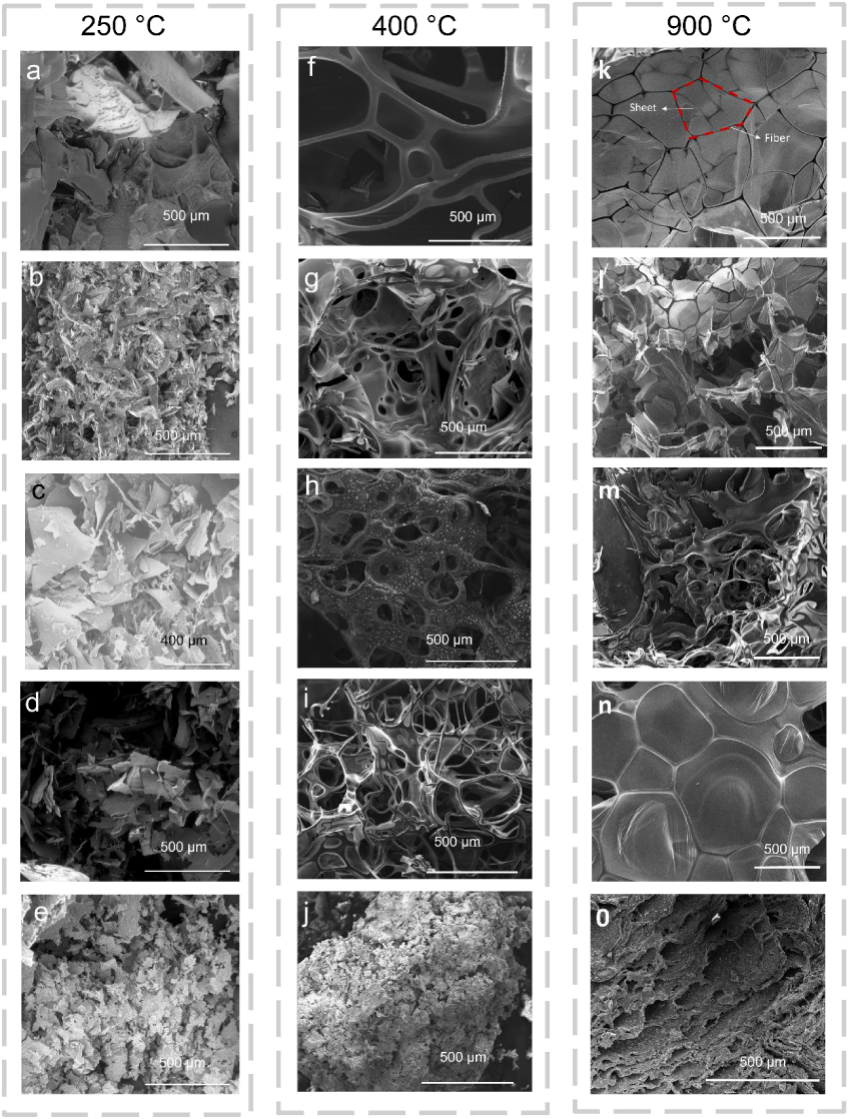
Structural morphology of HGAs prepared using various precursors at different peak pyrolysis temperatures. SEM images of samples prepared from PEW, α-lactalbumin, β-lactoglobulin, BSA, and yogurt after pyrolysis at (a–e) 250 °C, (f–j) 400 °C, and (k–o) 900 °C, respectively.

The morphological evolution observed across the temperature range reflects the progressive transformation of the protein precursors from organic-rich residues to carbonized frameworks. Between 200 and 450 °C, extensive mass loss occurs as volatile species are released, and the softened precursor displays a char-like viscous state.^15,21^ Gas evolution within this viscous char-like residue generates internal bubbles whose growth and coalescence govern the development of sheet- and fiber-like structures, as seen in PEW, α-lactalbumin, β-lactoglobulin and BSA derived samples. During this stage, the interplay between internal gas pressure, surface tension, and viscosity in the char-like residue determines the stability of the expanding bubbles.^15^ The continuous release and rupture of gases lead to local collapse and reconsolidation, resulting in partially developed and irregular sheets at 400 °C. As the temperature increases to 900 °C, the progressive carbonization of the matrix reduces mobility and locks the foamed structure in place, yielding well-defined sheets and fibers that preserve the morphology of the arrested foamed state. As demonstrated in our prior work, variations in thermal protocols such as heating rate can modulate protein decomposition kinetics and gas-release time scales during pyrolysis.^15^ These changes directly influence the balance between gas release and foaming effect, enabling systematic control over aerogel microstructural features such as fiber thickness, density, and porosity. Slower heating rates allow greater time for gas diffusion and mass redistribution, resulting in thicker structural elements and denser networks, whereas faster heating rates promote rapid expansion and the formation of thinner fibers and highly porous architectures.^15^ These microstructural variations, in turn, govern material properties, including compressive mechanical response, with denser networks exhibiting higher stiffness and strength and more porous structures favoring lightweight, compliant behavior.

In contrast, the yogurt-derived sample displays a distinct morphology; even at 900 °C, the structure remains irregular, rough, and porous, without the development of interconnected sheets or fiber-like structures. Density and porosity measurements reveal a clear distinction between pristine protein (PEW) and fat-rich precursor (yogurt) as summarized in the Supplementary Table 1, with the former yielding ultralow-density (3.62 mg/cm^3^), highly porous aerogels (99.83%) and the latter producing dense (195.20 mg/cm^3^), relatively less porous (91.12%) carbon structures.^15^ Although all precursors originate from similar protein sources, compositional variations such as the presence of fat and minerals in yogurt are likely to alter the rheological behavior of the precursor during heating. These components can modify surface tension and viscosity, impede bubble growth and coalescence, and thereby suppress the self-foaming mechanism responsible for sheet and fiber formation.

To further investigate the behavior of the yogurt precursor, a control experiment was conducted to isolate the effect of nonprotein constituents such as fats. Yogurt contains approximately 50% protein, with the remaining fraction composed of fats and inorganic species.^22^ The protein component primarily consists of casein and whey in a 4:1 ratio. To replicate the protein fraction without the additional components, a mixture of casein and whey in the same ratio was prepared and subjected to pyrolysis under identical conditions. The resulting carbon structure, shown in Supplementary Figure S2, exhibited the formation of sheet- and fiber-like morphologies similar to those observed for the other protein precursors. This confirms that the protein fraction alone can undergo the self-foaming transformation during pyrolysis and that the presence of fats and inorganic species in yogurt likely inhibits this process. The suppressive effect of fats on foaming is consistent with previous findings that fats influence the strength of protein gels formed during thermal denaturation by increasing network rigidity and interfacial cohesion.^23^ Here, the role of minerals is inferred from compositional comparisons of readily available precursors rather than from isolated mineral addition, and focused investigation employing controlled mineral addition would provide additional mechanistic resolution and is identified as an important direction for future work aimed at tuning structure and functionality. These results further suggest that the presence of fats alters the viscoelastic properties of the precursor during pyrolysis, suppressing bubble growth and preventing the formation of the interconnected sheet and fiber morphology observed in the other protein-derived aerogels.

The elemental composition was examined to gain further insight into the chemical characteristics of the samples formed from different protein-based precursors at 900 °C. The energy-dispersive X-ray spectroscopy (EDS) results, presented in Figure 3, show that most of the protein-derived aerogels are primarily composed of carbon (∼90%) and oxygen (∼9%), with minor traces of elements such as sodium, magnesium, sulfur, potassium, phosphorus, and calcium. In contrast, the carbon sample synthesized from yogurt exhibits a substantially different composition, containing 53.8% C, 28.3% O, 9.7% Ca, and 5.9% P, along with small amounts of K, Mg, and S. The presence of significant amount of inorganic constituents such as calcium may also modify thermal decomposition behavior, disrupting the balance between precursor softening and gas evolution during pyrolysis and thereby hindering the development of the sheet- and fiber-like morphology.

**Figure 3:**
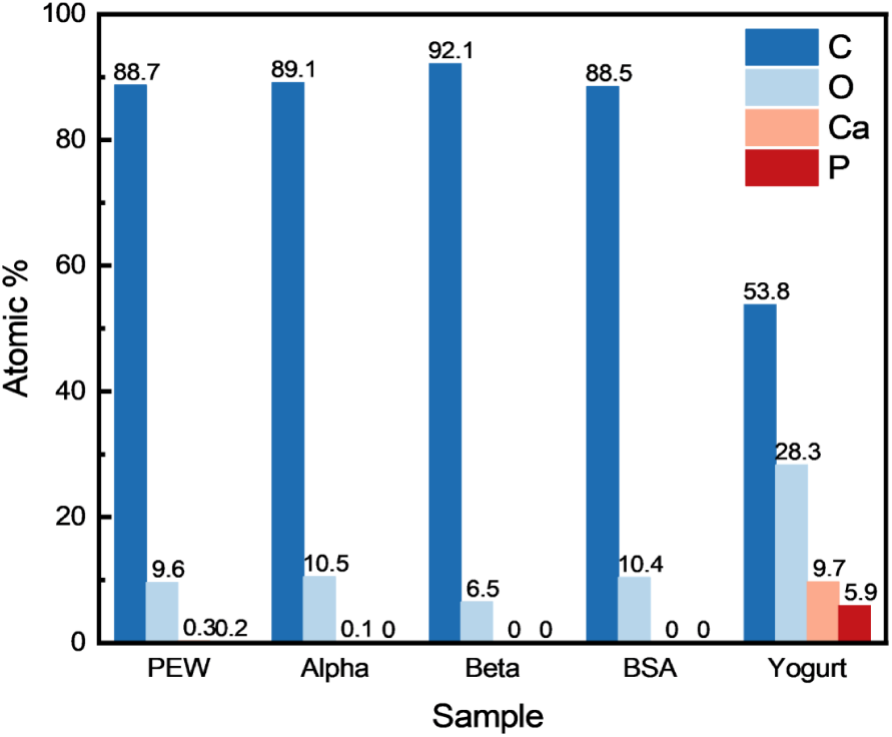
Chemical composition of samples prepared from various precursors pyrolyzed at a peak processing temperature of 900 °C.

Raman spectroscopic analysis was performed to evaluate the degree of structural ordering in the aerogels synthesized at 400 and 900 °C (Figure 4). Across all samples, two characteristic bands are observed at approximately 1337 cm^–1^ and 1590 cm^–1^, corresponding to the D and G peaks, respectively.^24^ The G band arises from the E_2g_ vibrational mode of sp^2^-hybridized carbon atoms, whereas the D band is attributed to defect-induced scattering associated with sp^3^-hybridized carbon or structural distortions. The relative intensity and sharpness of these peaks therefore provide insight into the level of disorder and the extent of graphitic ordering. At 400 °C, the Raman spectra of all samples display broad and overlapping D and G bands with shallow valleys between them, indicative of highly disordered carbonaceous structures.^25^ This behavior reflects the incomplete carbonization and the presence of amorphous intermediates at this temperature. In contrast, the spectra of samples pyrolyzed at 900 °C show relatively defined D and G peaks with a deeper valley between them, signifying improved ordering and growth of sp^2^ carbon domains.

**Figure 4:**
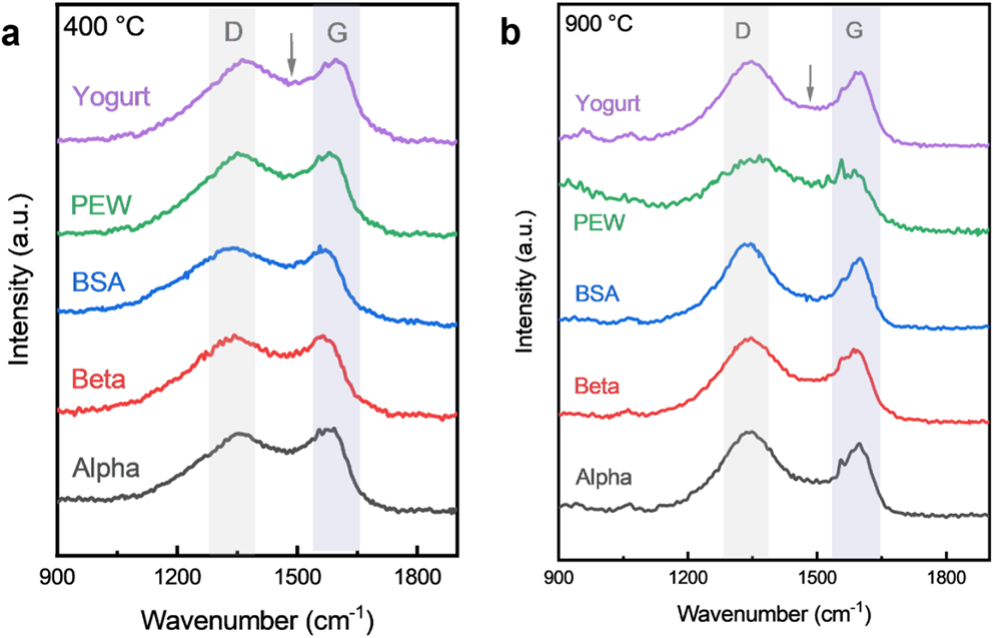
Raman analysis of samples prepared at the peak processing temperature of (a) 400 °C and (b) 900 °C.

Protein-derived graphitic aerogels are of interest for a range of applications due to their interconnected porous architecture, low density, and continuous graphitic carbon framework. This material system has been demonstrated for water purification, where hierarchical porosity enables efficient mass transport and residual heteroatom functionality contributes to salt and microplastic adsorption, achieving microplastic removal efficiencies of up to 99.9%, salt removal efficiencies of approximately 98%, and salt adsorption capacities on the order of ∼30 g/g.^14^ Related porous carbons produced through similar processing routes have also been shown to function as effective photothermal agents for plastic recycling, enabling the depolymerization of a broad range of polymers, including vinyl polymers, polyesters, and polycarbonates, back into their monomers with yields approaching 99% and sustained performance over multiple reuse cycles, highlighting their potential in recycling applications.^16^ Infrared thermography heat-shielding measurements were used as a demonstration of thermal insulation behavior (Supplementary Figure S4). After 300 s of heating, protein-rich PEW-derived aerogels exhibited substantially larger temperature difference between the hot plate and the sample surface than dense reference carbon materials. Specifically, the PEW-derived aerogel showed a lower surface temperature by ∼30% relative to the hot-plate temperature for a 3 mm thick sample and ∼43% for a 5 mm thick sample, compared with ∼24% for yogurt-derived carbon and ∼16% for graphite (dense) under similar conditions. The enhanced thermal insulation with increasing porosity and thickness is consistent with suppressed heat transfer in ultralow-density, highly porous architectures and provides an initial structure–property correlation linking precursor-dependent morphology to application-relevant thermal response. Together, these demonstrations provide context for the present work and highlight the importance of developing this material system from a wide range of precursors for these important applications.

From a scalability and commercialization perspective, the present approach benefits from the use of abundant protein feedstocks. Prior studies on porous graphitic aerogels and three-dimensional graphene analogues have demonstrated that synthesis routes relying on external chemical agents or templating strategies can achieve scalable production but often involve additional material and processing costs. For example, sugar-blowing–derived graphene aerogels have been reported with material costs on the order of ∼$0.5 g^–1^ at the laboratory scale.^21^ In contrast to such approaches, the method reported here does not require external foaming agents or templating additives, as self-foaming arises intrinsically from protein decomposition during pyrolysis. This suggests that graphitic aerogels produced from protein-based feedstocks offer a favorable route for achieving cost-competitive production relative to existing self-foaming porous carbon systems.

## Conclusions

This study investigates the formation of HGAs from various protein-based precursors and shows that precursor composition influences microstructure development during pyrolysis. PEW, α-lactalbumin, β-lactoglobulin, and BSA yield interconnected sheet and fiber-like networks, while the yogurt-derived samples possess rough and porous structures with no sheet or fiber features. These contrasting morphologies arise from compositional differences that likely influence decomposition kinetics, surface tension, and viscoelastic behavior during pyrolysis. In the protein systems, gas evolution occurs concurrently with precursor softening, producing a foaming effect that develops continuous hierarchical carbon networks upon carbonization. In contrast, the presence of substantial amounts of fats and mineral constituents such as calcium in the yogurt prevents foaming. Pyrolysis of a casein–whey mixture (a major component of yogurt) produces sheet and fiber-like structures, indicating that additional components in yogurt inhibit the foaming process. Raman analysis shows enhanced structural order from 400 to 900 °C, indicated by narrower D and G bands and a deeper valley between them. Overall, this work provides insight into developing HGAs from diverse protein precursors and shows how precursor composition influences microstructural evolution during protein pyrolysis, offering valuable guidance for the design of HGA-based composites and functional materials.

## Supplementary Material



## Notes

Details on DSC of different protein precursors; SEM image of the casein/whey mixture–derived sample; density and porosity data of PEW- and yogurt-derived samples; TGA analysis of the precursor (PEW) up to 900 °C (PDF)

## References

[1] Wu M., Geng H., Hu Y., Ma H., Yang C., Chen H., Wen Y., Cheng H., Li C., Liu F., Jiang L., Qu L. (2022). Superelastic Graphene Aerogel-Based
Metamaterials. Nat. Commun..

[2] Sun Z., Fang S., Hu Y. H. (2020). 3D Graphene
Materials: From Understanding
to Design and Synthesis Control. Chem. Rev..

[3] Li C., Yang J., Pachfule P., Li S., Ye M.-Y., Schmidt J., Thomas A. (2020). Ultralight Covalent Organic Framework/Graphene
Aerogels with Hierarchical Porosity. Nat. Commun..

[4] Sultanov F., Mentbayeva A., Kalybekkyzy S., Zhaisanova A., Myung S.-T., Bakenov Z. (2023). Advances of
Graphene-Based Aerogels
and Their Modifications in Lithium-Sulfur Batteries. Carbon.

[5] Pottathara Y. B., Tiyyagura H. R., Ahmad Z., Sadasivuni K. K. (2020). Graphene
Based Aerogels: Fundamentals and Applications as Supercapacitors. J. Energy Storage.

[6] Nassar G., Daou E., Najjar R., Bassil M., Habchi R. (2021). A Review on
the Current Research on Graphene-Based Aerogels and Their Applications. Carbon Trends.

[7] Wang H., Mi X., Li Y., Zhan S. (2020). 3D Graphene-Based Macrostructures
for Water Treatment. Adv. Mater..

[8] Mu Y., Wang L., Zhang R., Pashameah R. A., Alzahrani E., Li Z., Alanazi A. K., Algadi H., Huang M., Guo Z., Wan T., Wei H. (2023). Rapid and
Facile Fabrication of Hierarchically Porous Graphene Aerogel for Oil-Water
Separation and Piezoresistive Sensing Applications. Appl. Surf. Sci..

[9] Deng X., Nie Q., Wu Y., Fang H., Zhang P., Xie Y. (2020). Nitrogen-Doped
Unusually Superwetting, Thermally Insulating, and Elastic Graphene
Aerogel for Efficient Solar Steam Generation. ACS Appl. Mater. Interfaces.

[10] Li F., Zhu J., Sun P., Zhang M., Li Z., Xu D., Gong X., Zou X., Geim A. K., Su Y., Cheng H. M. (2022). Highly Efficient
and Selective Extraction of Gold by
Reduced Graphene Oxide. Nat. Commun..

[11] Zhu M., Kong L., Xie M., Lu W., Liu H., Li N., Feng Z., Zhan J. (2021). Carbon Aerogel from Forestry Biomass
as a Peroxymonosulfate Activator for Organic Contaminants Degradation. J. Hazard. Mater..

[12] Wang Y., Liu R., Tian Y., Sun Z., Huang Z., Wu X., Li B. (2020). Heteroatoms-Doped Hierarchical Porous Carbon Derived from Chitin
for Flexible All-Solid-State Symmetric Supercapacitors. Chem. Eng. J..

[13] Sam D. K., Sam E. K., Durairaj A., Lv X., Zhou Z., Liu J. (2020). Synthesis
of Biomass-Based Carbon Aerogels in Energy and Sustainability. Carbohydr. Res..

[14] Ozden S., Monti S., Tozzini V., Dutta N. S., Gili S., Caggiano N., Link A. J., Pugno N. M., Higgins J., Priestley R. D., Arnold C. B. (2022). Egg Protein Derived Ultralightweight
Hybrid Monolithic Aerogel for Water Purification. Mater. Today.

[15] Wani M. S., Denzer B., Caggiano N. J., Prud’homme R. K., Arnold C. B. (2025). Hierarchically Porous Graphitic Aerogels via Thermal
Morphogenesis of Proteins for Environmental Remediation. ACS Appl. Nano Mater..

[16] Jang, Y.-J. ; Wani, M. S. ; Arnold, C. ; Stache, E. A Universal Approach for Chemical Recycling to Monomers Using Photothermally Activated Hierarchically Porous Carbon. ChemRxiv, 2025, 10.26434/chemrxiv-2025-cl4ld.

[17] Hayashi S., Das A., Rupp M., Wani M. S., Arnold C. B. (2025). Freeform Monolithic
Graphitic Aerogels by Laser Pyrolysis of Pretreated Blood-Derived
Feedstocks. Matter.

[18] Sun Y., Xu D., He Z., Zhang Z., Fan L., Wang S. (2023). Green Fabrication
of Pore-Modulated Carbon Aerogels Using a Biological Template for
High-Energy Density Supercapacitors. J. Mater.
Chem. A.

[19] Azevedo V. M., Borges S. V., Marconcini J. M., Yoshida M. I., Neto A. R. S., Pereira T. C., Pereira C. F. G. (2017). Effect of Replacement of Corn Starch
by Whey Protein Isolate in Biodegradable Film Blends Obtained by Extrusion. Carbohydr. Polym..

[20] Cho S. Y., Yun Y. S., Lee S., Jang D., Park K.-Y., Kim J. K., Kim B. H., Kang K., Kaplan D. L., Jin H.-J. (2015). Carbonization of a Stable β-Sheet-Rich
Silk Protein
into a Pseudographitic Pyroprotein. Nat. Commun..

[21] Wang X., Zhang Y., Zhi C., Wang X., Tang D., Xu Y., Weng Q., Jiang X., Mitome M., Golberg D. (2013). Three-Dimensional
Strutted Graphene Grown by Substrate-Free Sugar
Blowing for High-Power-Density Supercapacitors. Nat. Commun..

[22] Sumi K., Tagawa R., Yamazaki K., Nakayama K., Ichimura T., Sanbongi C., Nakazato K. (2023). Nutritional
Value of Yogurt as a
Protein Source: Digestibility/Absorbability and Effects on Skeletal
Muscle. Nutrients.

[23] Aguilera J. M., Xiong Y. L., Kinsella J. E. (1993). Viscoelastic
Properties of Mixed
Dairy Gels. Food Res. Int..

[24] Li Z., Deng L., Kinloch I. A., Young R. J. (2023). Raman Spectroscopy
of Carbon Materials and Their Composites: Graphene, Nanotubes and
Fibres. Prog. Mater. Sci..

[25] Rigollet S., Béguerie T., Weiss-Hortala E., Flamant G., Nzihou A. (2025). Synthesis
of Graphitic Biocarbons from Lignin Fostered by Concentrated Solar
Energy. Sci. Rep..

