# Supplementary material for: Influence of Precursor Composition on Microstructure Formation in Protein-Derived Porous Graphitic Aerogels
Source: ACS Mater Au. 2026 Jan 27;6(3):576–82. doi: 10.1021/acsmaterialsau.5c00227 (PMC13177403; doi:10.1021/acsmaterialsau.5c00227)
Supplement: Supplementary file 1 [file mg5c00227_si_001.pdf]

Influence of Precursor Composition on Microstructure Formation in Protein-Derived Porous Graphitic Aerogels

M. Shaharyar Wani[1,2], Elizabeth G. Stump[1,2], Bridget R. Denzer[3], Craig B. Arnold[1,2*]

[1]Department of Mechanical & Aerospace Engineering, Princeton University, Princeton, NJ, 08544, USA

[2]Princeton Materials Institute, Princeton University, Princeton, NJ 08544, USA

[3]Department of Materials Science & Engineering, Massachusetts Institute of Technology, Cambridge, Massachusetts, 02139, USA

[*]Corresponding Author: C.B.A. cbarnold@princeton.edu

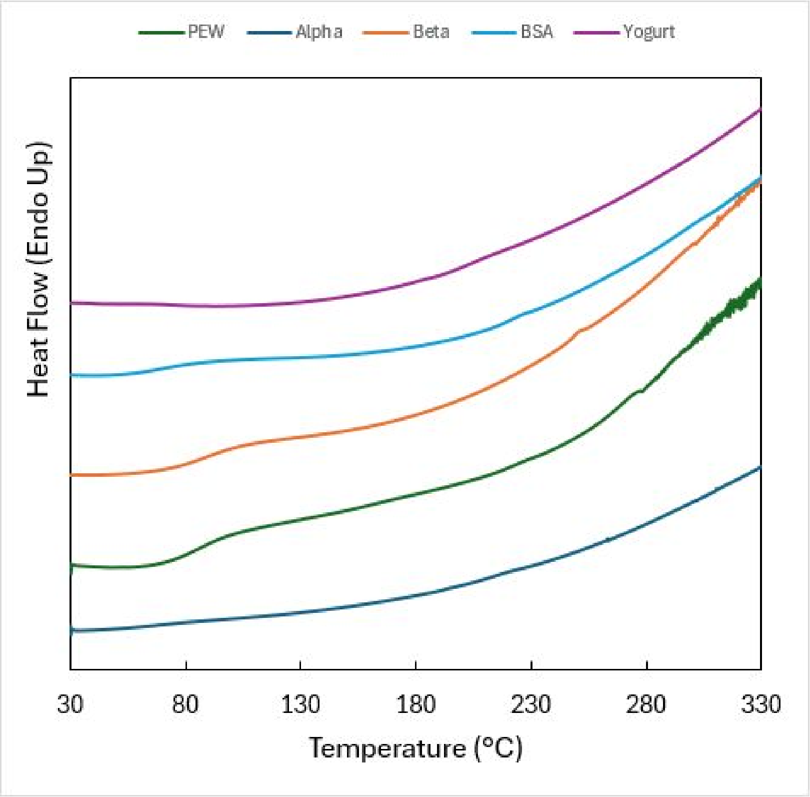

Supplementary Figure S1. DSC analysis of different proteins (PEW, α-lactalbumin, β-lactoglobulin, BSA and yogurt).

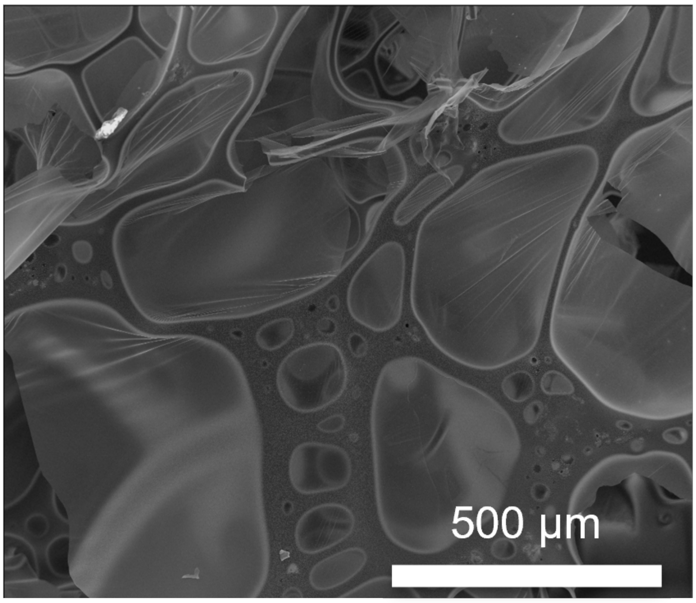

Supplementary Figure S2. Microstructure of HGA developed from a casein/whey mixture (4:1 ratio by mass).

Supplementary Table 1: Comparison of density and porosity of pristine protein and fat-rich protein.

Sample	Density (mg/cm^3)	Porosity (%)
Pristine protein (PEW)[1]	3.62	99.83
Fat and mineral-containing protein (Yogurt)	195.21	91.12

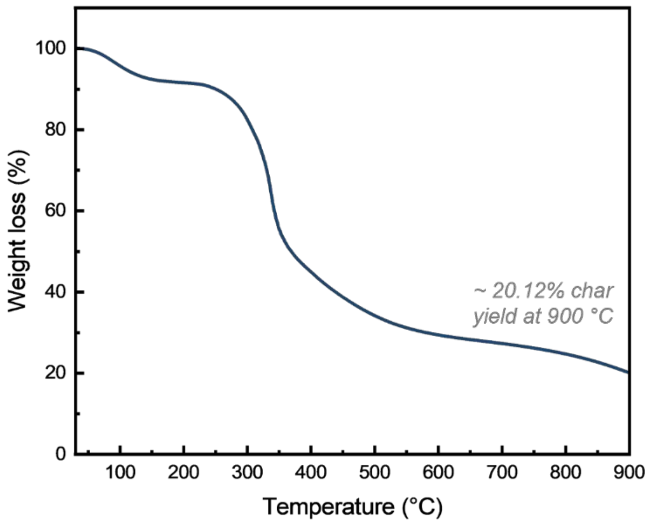

Supplementary Figure S3. Thermogravimetric analysis of the protein precursor (PEW) up to 900 °C under nitrogen, showing thermal decomposition behavior and char yield.

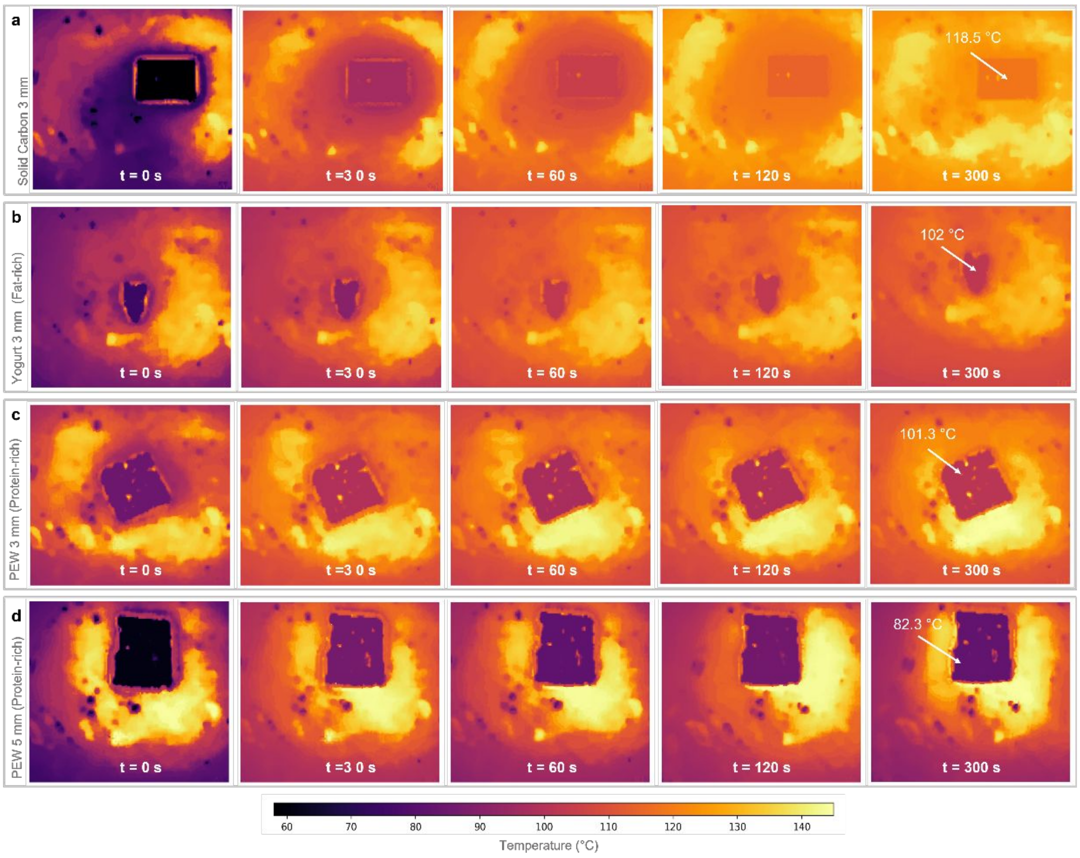

Supplementary Figure S4. Infrared thermography images of (a) dense carbon (graphite, 3 mm), (b) yogurt-derived carbon aerogel from a fat-rich precursor (3 mm), (c) PEW-derived

carbon aerogel from a protein-rich precursor (3 mm), and (d) PEW-derived carbon aerogel from a protein-rich precursor (5 mm).

References

(1) Wani, M. S.; Denzer, B.; Caggiano, N. J.; Prud'homme, R. K.; Arnold, C. B. Hierarchically Porous Graphitic Aerogels via Thermal Morphogenesis of Proteins for Environmental Remediation. *ACS Appl Nano Mater* 2025, 8 (16), 8464–8472. https://doi.org/10.1021/acsanm.5c01156.
